# Dl-3-n-Butylphthalide Reduced Neuroinflammation by Inhibiting Inflammasome in Microglia in Mice after Middle Cerebral Artery Occlusion

**DOI:** 10.3390/life12081244

**Published:** 2022-08-16

**Authors:** Mengdi Liu, Haoran Zheng, Ze Liu, Yiyan Guo, Shuhong Wang, Yaohui Tang, Hengli Tian, Zhijun Zhang, Guoyuan Yang

**Affiliations:** 1 Med-X Research Institute, School of Biomedical Engineering, Shanghai Jiao Tong University, 1954 Hua Shan Road, Shanghai 200030, China; 2 Department of Neurology, Shanghai East Hospital, Shanghai 200123, China; 3 Department of Neurosurgery, Shanghai Jiao Tong University Affiliated Sixth People’s Hospital, Shanghai Jiao Tong University, Shanghai 200233, China

**Keywords:** dl-3n-butylphthalide, ischemic stroke, microglia, NLRP3 inflammasome

## Abstract

The inflammatory response is one of the key events in cerebral ischemia, causing secondary brain injury and neuronal death. Studies have shown that the NLRP3 inflammasome is a key factor in initiating the inflammatory response and that Dl-3-n-butylphthalide (NBP) can attenuate the inflammatory response and improve neuronal repair during ischemic stroke. However, whether NBP attenuates the inflammatory response via inhibition of NLRP3 remains unclear. A 90 min middle cerebral artery occlusion was induced in 62 2-month-old adult male ICR mice, and NBP was administered by gavage zero, one, or two days after ischemia. Brain infarct volume, neurological deficits, NLRP3, microglia, and neuronal death were examined in sacrificed mice to explore the correction between NBP effects and NLRP3 expression. NBP significantly reduced infarct volume and attenuated neurological deficits after ischemic stroke compared to controls (*p* < 0.05). Moreover, it inhibited ASC^+^ microglia activation and NLRP3 and CASP1 expression in ischemic mice. In addition, neuronal apoptosis was reduced in NBP-treated microglia cultures (*p* < 0.05). Our results indicate that NBP attenuates the inflammatory response in ischemic mouse brains, suggesting that NBP protects against microglia activation via the NLRP3 inflammasome.

## 1. Introduction

Stroke is a leading cause of death and disability worldwide [1], causing a sharp decline in the quality of life of patients and a heavy burden on society. After a stroke, the brain suffers many neuronal cell deaths and complex pathological changes [2]. Ischemic stroke is the most common type of stroke, resulting in reduced blood flow to the brain, oxidative stress, metabolic imbalance, neuronal cell death, and local inflammatory response [3]. Current acute ischemic stroke treatments include intravenous thrombolytic therapy with recombinant tissue plasminogen activator (rtPA) and intravascular therapy [4,5]. However, rapid blood flow reperfusion after thrombolysis enhances ROS production, exacerbating brain tissue damage.

The inflammatory response is important in the acute phase of a stroke. Microglia are the primary inflammatory cells in the brain, continuously surveying the central nervous system (CNS) environment and quickly responding to the stimuli by releasing multiple cytokines and inflammation factors [6], including tumor necrosis factor (TNF) and interleukins 1 (IL-1) and 6 (IL-6), which disrupt the blood-brain barrier (BBB) and exacerbate brain edema and micro-bleeding [7,8]. The release of inflammatory cytokines also exacerbates ischemic brain injury.

Recent studies identified a unique inflammatory factor, the NLR family pyrin domain containing 3 (NLRP3) inflammasome, which is an important mediator of cell damage and inflammation after ischemic stroke [9]. Microglia are the largest producers of the NLRP3 inflammasome [10]. NLRP3 inflammasome activation requires two conditions: (1) NLRP3 transcription surpasses a certain threshold; (2) NLRP3 recruitment of apoptosis-associated speck-like protein containing a CARD (ASC) and caspase 1 (CASP1) [11]. In the acute phase of ischemic stroke, molecules released by necrotic cells represent damage-associated molecular patterns (DAMPs) [12]. When recognized by microglia, DAMPs can induce nuclear factor kappa-light-chain-enhancer of activated B cells (NF-κB) and *NLRP3* expression, inducing the inflammatory response [13]. Studies have found increased NLRP3 inflammasome expression in brain tissue after cerebral ischemia [14]. After NLRP3 inflammasome assembly, CASP1 activation promotes interleukin 1β (IL-1β) and 18 (IL-18) maturation and regulates pyroptosis by cleaving gasdermin D (GSDMD) [15]. The NLRP3 inflammasome is involved in the ischemic brain injury process, and its inhibition provides a novel therapeutic target for ischemic stroke [16,17].

Dl-3-n-butylphthalide (NBP) is a compound approved by the Chinese Food and Drug Administration to treat acute ischemic stroke [18], improving neurological function and reducing infarct volume, neuronal cell death, and BBB leakage [19,20,21,22]. In addition, NBP reduced the inflammatory response in Alzheimer’s disease by inhibiting microglial NLRP3 inflammasome activation [23]. Moreover, NBP reduced NLRP3, CASP1, and IL-1β protein levels in ischemic stroke [24]. However, the effect of NBP on the NLRP3 inflammasome during and after ischemic stroke remains unknown.

In this study, we explored the effects of NBP on the microglial NLRP3 inflammasome after cerebral ischemia in transient middle cerebral artery occlusion (tMCAO) mice.

## 2. Materials and Methods

### 2.1. Experimental Design

This study was approved by the Institutional Animal Care and Use Committee of Shanghai Jiao Tong University, Shanghai, China. The mouse experiments were performed in accordance with the Reporting of in vivo Experiments (ARRIVE) guidelines. The mice were housed under a standard 12 h light/dark cycle and at a constant temperature of 25 ± 1 °C, with free access to food and water throughout experiments. All experiments were performed to minimize suffering by the mice. In this case, 62 adult male ICR mice (body weight 26 ± 2 g) were randomly divided into three groups: (1) NBP treated (*n* = 22); (2) oil treated control (*n* = 24); and (3) sham (*n* = 10). The sample size was determined based on our previous paper, the results of our preliminary experiment and the “Powerandsamplesize.com” website (accessed on 25 February 2020) [25]. The minimum sample size of each group calculated based on the cerebral infarction volume was three. Considering sample loss during the experiment, we increased the sample size of each group to 3–4. NBP or oil was given by gavage immediately after tMCAO, and additional doses were given once daily on days 1 and 2 after tMCAO. Modified neurological severity score (mNSS) and body weight were measured in the first three days after tMCAO. Six mice died during or after the tMCAO procedure. In this case, 32 mice were sacrificed on day 3 for the brain tissue analysis. The other 24 mice were used for neurobehavioral tests on days 7 and 14. Most analyses were performed blind.

### 2.2. Establishment of the tMCAO Mouse Model

tMCAO is a focal cerebral ischemia model, the most widely used cerebral ischemia model at present. The tMCAO adhered to previously reported experimental procedures [26]. First, mice were anesthetized with 4% isoflurane, which was maintained with 1.5% isoflurane throughout the surgical procedure. Mice were placed on the operating table with their necks facing the surgical microscope. The skin of the neck was opened, and the common (CCA), internal (ICA), and external (ECA) carotid arteries were explored. A silica-coated 6-0 nylon monofilament suture (Covidien; Minneapolis, MN, USA) was gently inserted from the ECA into the ICA until it blocked the MCA opening. Blood flow was monitored by a laser Doppler flowmetry (Moor Instruments, Devin, UK) before and after tMCAO to ensure the tMCAO model reproducibility. After 90 min of ischemia, the suture was removed. Occlusion success was evaluated based on ipsilateral blood flow reduced to 10% of the contralateral blood flow. Reperfusion success was considered ipsilateral blood flow recovery to >80% of the contralateral blood flow. The water and food consumption of mice after surgery was monitored. Six mice were excluded due to death or extreme discomfort during or after the post-surgery observational period. A lethal dose of isoflurane was used to euthanize the mice.

### 2.3. Neurobehavioral Tests Determination

The mNSS and rotarod test were performed blind. On days 1, 3, 7, and 14 after tMCAO, mNSS was used to assess the neurological outcomes and included motor, balance, and reflex tests. The total mNSS was 12 (normal score = 0; maximal deficit score = 12). The higher the score, the more severe the neurological deficit [27]. The rotarod test required training three days before tMCAO. The mice were examined on days 7 and 14 after tMCAO. The residence time on the rotarod machine at 40 rpm was recorded [28].

### 2.4. Brain Infarct Volume Measurement

Mice were sacrificed on day 3 after tMCAO. Brain tissue was taken after phosphate-buffered saline (PBS) perfusion and stored at −80 °C. Frozen brain sections were cut at 20 μm thickness at an interval of 200 μm. Brain sections were stained with 0.1% cresyl violet solution (Meilun Chemical Reagent Co.; Liaoning, China). The Image J software (US National Institutes of Health [NIH]; Bethesda, MD, USA; RRID: SCR_003070) was used to measure the contralateral and ipsilateral non-infarct areas. The infarct volume was calculated with the following equation:

$$V=\overset{n}{\underset{1}{\sum }}\frac{H\left[\Delta Sn+\Delta S\left(n+1\right)+\sqrt{\Delta Sn+\Delta S\left(n+1\right)}\right]}{3}$$

Here, the *H* represents the distance between the two brain slices (200 µm), and *S* represents the difference between the contralateral and ipsilateral non-infarct areas.

### 2.5. Immunohistochemical Staining

Brain sections were fixed in 4% paraformaldehyde (PFA) for 10 min, and the cell membrane was disrupted with 0.3% Triton X-100 for 10 min. Blocking was performed with 1% bovine serum albumin (BSA) for 1 h. Next, brain sections were incubated with antibodies against cluster of differentiation 11b (CD11b; 1:200, Cell Signaling Technology; Boston, MA, USA), CASP1 (1:200, Santa Cruz Biotechnology; Paso Robles, CA, USA), or ASC (1:200, Cell Signaling Technology) at 4 °C for 16 h. The sections were washed thrice with PBS and incubated at room temperature for 1 h with a secondary antibody corresponding to the primary antibody. The secondary antibody was washed off with PBS and stored at −20 °C after adding an antifade mounting medium containing 4′,6-diamidino-2-phenylindole (DAPI; Life Technologies; Mulgrave, Australia). Deoxynucleotidyl transferase dUTP nick end labeling (TUNEL) staining was performed with a one-step TUNEL apoptosis assay kit (Meilunbio; Shanghai, China). Stained sections were observed with the TCS SP5 Confocal Scanning System (Leica; Wetzlar, Germany). Four fields along the peri-infarct regions were selected for each section.

### 2.6. Western Blotting Analysis

Fresh mouse brains were collected and sliced into 2 mm sections in a mouse brain mold (RWD Company; Shanghai, China). We chose the ipsilateral cerebral hemisphere (2 mm posterior to the bregma), including the cortex and striatum, for Western blotting. Proteins were separated on a 10% sodium dodecyl sulfate (SDS)-polyacrylamide gel (PAGE) and then transferred to a polyvinylidene fluoride (PVDF) membrane. The membrane was blocked in the protein-free rapid blocking buffer (EpiZyme; Shanghai, China) for 15 min. Then, membranes were incubated with primary antibodies against NLRP3 (1:1000; Cell Signaling Technology), ASC (1:1000; Cell Signaling Technology), CASP1 (1:1000; Santa Cruz Biotechnology), β-actin (ACTB; 1:1000; Santa Cruz Biotechnology) for 16 h at 4 °C. Next, membranes were thrice washed with tris-buffered saline containing 0.1% Tween 20 (TBST) buffer for 15 min and incubated with secondary antibodies conjugated with horseradish peroxidase (HRP) at room temperature for 1 h. The membranes were visualized using an enhanced chemiluminescence kit (ECL; Pierce; Waltham, MA, USA). Protein bands were quantified with the Image J. software.

### 2.7. Real-Time PCR

After tMCAO, fresh mouse brains were collected and sliced into 2 mm sections in a mouse brain mold. We chose the ipsilateral cerebral hemisphere (2 mm posterior to the bregma), including the cortex and striatum, for real-time quantitative reverse transcription PCR (RT-qPCR). TRIzol (Invitrogen; Carlsbad, CA, USA) was used to isolate total RNA according to the manufacturer’s instructions. A NanoDrop 1000 spectrophotometer (Thermo Fisher Scientific; Waltham, MA, USA) was used to quantify the isolated total RNA and confirm RNA concentration standardization across samples. Total RNA was reverse transcribed into complementary DNA (cDNA) using a cDNA synthesis kit (Yeasen; Shanghai, China). RT-qPCR was performed with SYBR Green Master Mix (Yeasen) according to the manufacturer’s instructions using the primer sequences listed in Table 1. The relative expression of genes was calculated with the 2^−ΔΔCT^ method.

### 2.8. Cell Culture

The primary culturing of neurons was performed as previously described [29]. Briefly, neurons were isolated from the cerebral cortex of an embryonic fetus (JSJ; Shanghai, China) and maintained in a humidified incubator at 37 °C and 5% CO_2_. BV2 cell culture medium was prepared with 5% fetal bovine serum (FBS; Gibco; Grand Island, NY, USA) and 1% penicillin-streptomycin dissolved in Dulbecco’s modified Eagle’s medium (DMEM; Gibico). Neurons were cultured in neurobasal-A medium supplemented with 2% B27.

### 2.9. Oxygen-Glucose Deprivation (OGD)

Cultured BV2 cells and neurons were rinsed twice with PBS, resuspended in DMEM without glucose, and immediately cultured in an anaerobic chamber containing 95% N_2_ and 5% CO_2_ for 1.5 h. Next, BV2 cells were resuspended in complete medium containing different NBP concentrations and incubated under aerobic conditions. Then, BV2 cells were collected for subsequent experiments. The culture medium of neurons was replaced with a 1:1 mixture of complete neuron and microglia cell medium. Neurons were collected for subsequent experimental analysis after 24 h.

### 2.10. Cell Proliferation and Cytotoxicity Assay

BV2 cells were seeded in 96-well plates with 10,000 cells per well and incubated with different NBP concentrations (10, 30, 50, 70, and 90 μmol/L) for 24 h. The cell counting kit 8 (CCK-8) was used to quantify cell proliferation and cytotoxicity. Briefly, 10 μL of CCK-8 solution was added to each well and incubated at 37 °C for 4 h. Then, the absorbance at 450 nm was measured with a microplate reader.

### 2.11. Statistical Analysis

Statistical analyses were performed with PASW Statistics for Windows v.18.0 (SPSS, Inc.; Chicago, IL, USA). Data distributions were compared with the Kolmogorov-Smirnov test. Intergroup comparisons were performed with unpaired Student’s *t*-tests or one-way analysis of variance (ANOVA). All data are represented as mean ± standard deviation (SD). Results with *p* < 0.05 were considered statistically significant.

## 3. Results

### 3.1. NBP Reduced Infarct Volume and Neurological Behavior in tMCAO Mice

**Figure 1:**
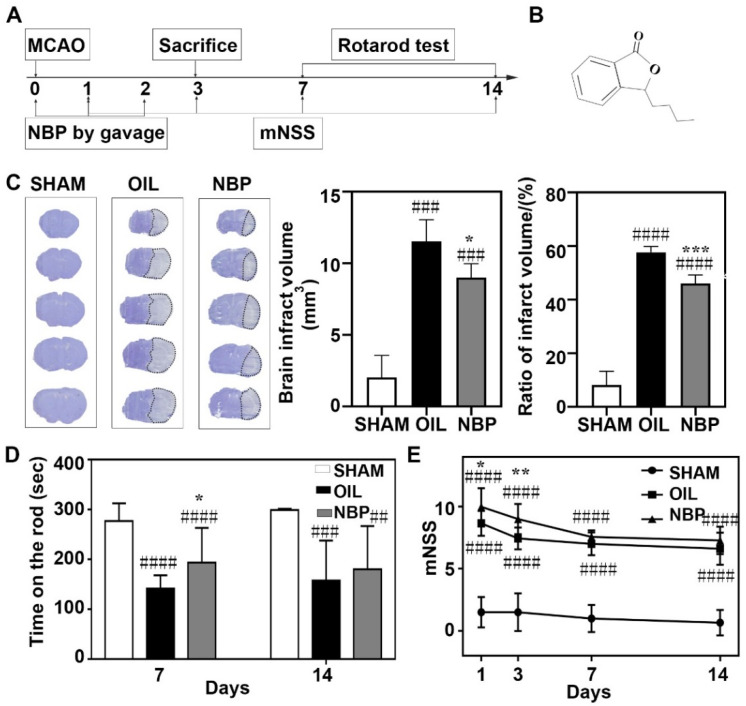
NBP attenuated neurological deficit and brain injury in tMCAO mice. (**A**) Flow chart of the experimental scheme. (**B**) Chemical structural formula of NBP. (**C**) Left: Cresyl violet staining of frozen section at day 3 after tMCAO in the NBP treated mice compared to the oil treated control mice. Right: Bar graphs showed the infarct volume and the percentage of infarct volume/contralateral hemisphere volume in the NBP treated mice compared to the oil treated control mice. (**D**) Bar graph showed that the rotarod test at day 7 and 14 after tMCAO in the SHAM, NBP treated and oil treated control groups. (**E**) Line graph showed that mNSS in the SHAM, NBP treated and oil treated control mice at 1, 3, 7 and 14 days after tMCAO. Data were presented as mean ± SD. N = 4 in Cresyl violet staining. N = 7–8 in behavior test. * *p* < 0.05, ** *p* < 0.01 and *** *p* < 0.001 compared to OIL group. ## *p* < 0.01, ### *p* < 0.001 and #### *p* < 0.0001 compared to SHAM group.

To assess the neuroprotective effect of NBP during the acute stage after tMCAO in mice, we examined the infarct volume and neurobehavioral outcomes after tMCAO (Figure 1A,B). We found that cerebral infarction volumes in the NBP-treated group decreased on day 3 after tMCAO (*p* < 0.05; Figure 1C). Rotarod tests, used to assess motor function, showed that the mice stayed longer on rotarod in the NBP-treated group compared to the control group (*p* < 0.05; Figure 1D). The mNSS showed that NBP treatment attenuated neurological deficits significantly on days 1 and 3 after tMCAO (*p* < 0.05; Figure 1E). These results show that NBP has a neuroprotective effect after tMCAO in mice.

### 3.2. NBP Attenuated the Inflammatory Factor Expression after tMCAO

**Figure 2:**
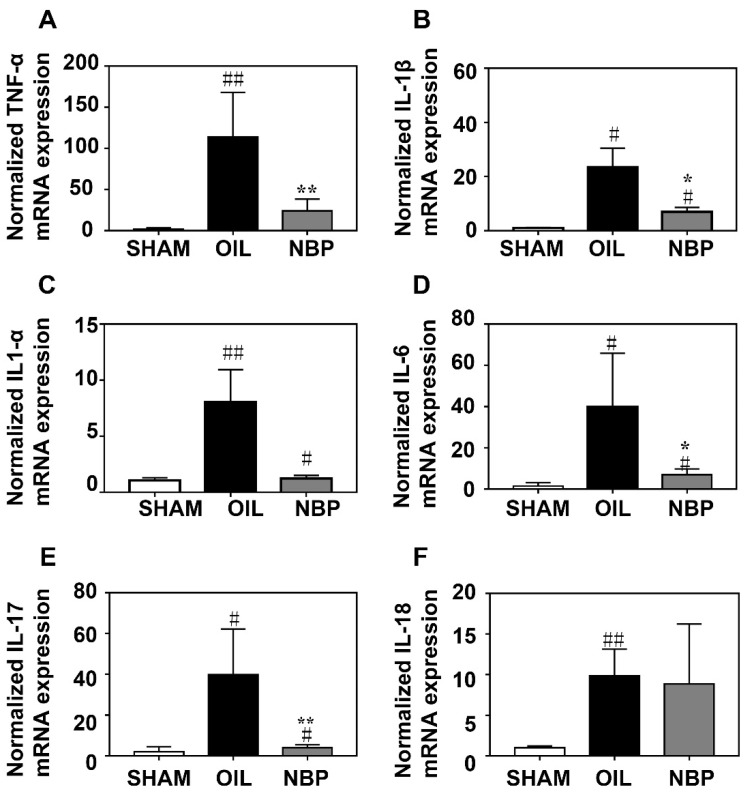
NBP decreased the inflammatory factor mRNA expression. Bar graphs showed that RT-PCR quantification of inflammatory factors TNF-α (**A**), IL-1β (**B**), IL-1α (**C**), IL-6 (**D**), IL-17 (**E**) and IL-18 (**F**) in the mouse brain after 3 days of tMCAO. N = 3–4 in each group. Data were presented as mean ± SD. * *p* < 0.05 and ** *p* < 0.01 compared to OIL group. # *p* < 0.05 and ## *p* < 0.01 compared to SHAM group.

NBP has anti-inflammatory effects in neurodegenerative diseases [30]. To determine whether NBP inhibited inflammatory responses during the acute phase of tMCAO, we quantified the mRNA levels of related pro-inflammatory factors tumor necrosis factor α (TNF-α), interleukin 1α (IL-1α), IL-1β, IL-6, interleukin 17 (IL-17), and IL-18 in the ipsilateral hemisphere brain samples after tMCAO. We found that TNF-α, IL-1β, IL-6, and IL-17 mRNA levels in the NBP-treated group were significantly lower on day 3 after tMCAO compared to the control group (*p* < 0.05, Figure 2A,B,D,E).

### 3.3. NBP Inhibited NLRP3 Inflammasome Expression in Microglia

**Figure 3:**
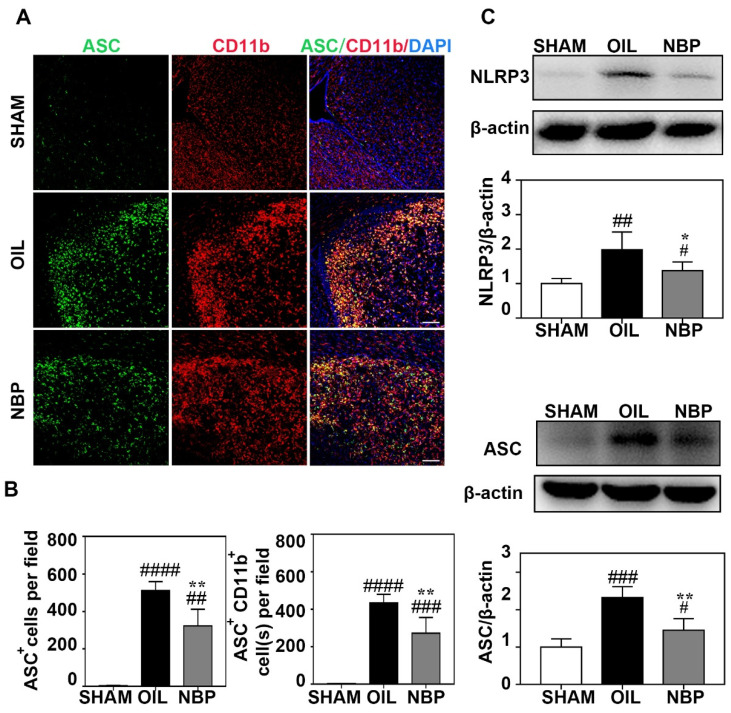
NBP inhibited the expression of inflammasome in microglial cells. (**A**) Photomicrographs showed that double immunostaining of ASC (green) and CD11b (red) in the NBP treated, the oil treated control, and sham mice at day 3 after tMCAO. (**B**) Bar graph showed that the numbers of ASC^+^ cells (left) and ASC^+^/CD11b^+^ cells (right) in the SHAM and NBP treated, the oil treated control goups of mice. (**C**) Photographs showed that Western blot bands of NLRP3 and ASC in the ipsilateral hemisphere of ischemic brain in the NBP treated, oil treated control, and sham mice. Bargraph showed that the intensity ratios of NLRP3/β-actin and ASC/β-actin in the NBP treated, oil treated groups and sham groups. Scale bar = 100 μm. N = 3–6 in each group. Data were presented as mean ± SD. * *p* < 0.05 and ** *p* < 0.01 compared to OIL group. # *p* < 0.05, ## *p* < 0.01, ### *p* < 0.001 and #### *p* < 0.0001 compared to SHAM group. Full pictures of the Western blots and the densitometry scans are presented in Figure S1.

Increasing evidence shows that NLRP3-inflammasome-mediated pyroptosis plays a critical role in inflammation [31]. The NLRP3 inflammasome is involved in the inflammatory response of ischemic stroke. To further explore the relationship between NBP treatment and inflammasome expression during the acute phase after tMCAO, we quantified NLRP3 and ASC protein levels in the ischemic brain. Immunostaining showed that numbers of ASC^+^ and ASC ^+^/CD11B^+^ cells were significantly lower in the ipsilateral hemisphere of the ischemic brain of NBP-treated mice than in control mice (*p* < 0.01; Figure 3A,B). In addition, NLRP3 and ASC protein levels were significantly lower on day 3 after tMCAO in NBP-treated mice than in control mice (*p* < 0.05; Figure 3C).

### 3.4. NBP Treatment Reduced CASP1 Expression in Neurons

**Figure 4:**
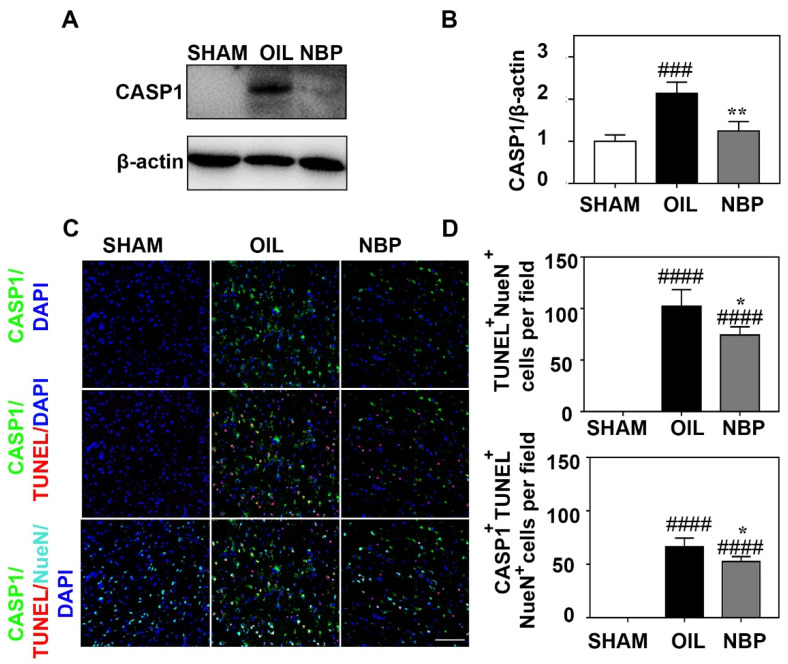
NBP inhibited the neuronal caspase-1 expression. (**A**) Representative images of Western blot analysis of caspse-1 expression in the NBP treated, oil treated control, and sham groups at the day 3 after tMCAO. (**B**) Bar graphes showed that CASP1 expression in the NBP treated, oil treated control, and sham groups at the day 3 after tMCAO. (**C**) Photomicrographes showed that triple immunostaining of CASP1 (green), TUNEL (red) and NeuN (cyan) in the NBP treated and the oil treated control groups at 3 days after tMCAO. (**D**) Bar graphs showed TUNEL^+^/NueN^+^ cells and caspase-1^+^/TUNEL^+^/NueN^+^ cells. Bar = 100 μm. N = 3–5 per group. Data were presented as mean ± SD. * *p* < 0.05, ** *p* < 0.01 compared to OIL group. ### *p* < 0.001 and #### *p* < 0.0001 compared to SHAM group. Full pictures of the Western blots and the densitometry scans are presented in Figure S2.

CASP1 is an important pro-inflammatory factor involved in pyroptosis. After NLRP3 recruits ASCs, CASP1 is cleaved to form cleaved-CASP1 [32]. To explore the effect of NBP on CASP1 during the acute phase after tMCAO, we quantified CASP1 levels in brain tissues from tMCAO mice. We found that CASP1 protein levels in the NBP-treated group were significantly lower than in the control group (*p* < 0.01; Figure 4A,B). To explore the relationship between neuronal death and *CASP1* expression after NBP treatment, we performed immunostaining. We found that the numbers of TUNEL^+^/NeuN^+^ and CASP1^+^/TUNEL^+^/NeuN^+^ cells in the perifocal region of the NBP-treated group were significantly lower than in the control group (*p* < 0.01; Figure 4C,D).

### 3.5. NBP Treatment Indirectly Reduced the Apoptosis of Neurons In Vitro

**Figure 5:**
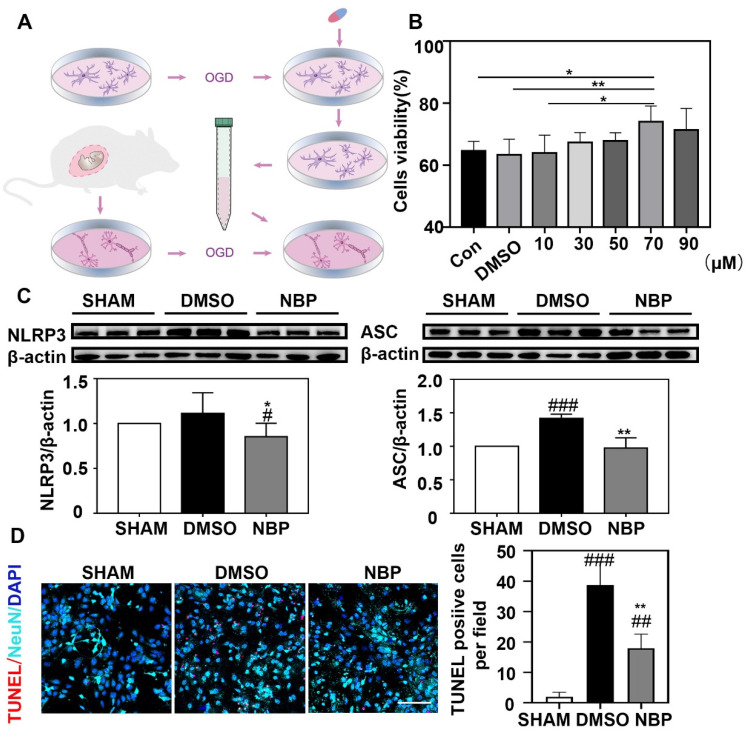
NBP could indirectly reduce the neuronal poptosis after OGD. (**A**) Flow chart of in vitro experiments. (**B**) Bar graph showed that the viability rate for BV2 cells treated with different concentrations of NBP after OGD. (**C**) Photographes showed that Western blot bands NLRP3 (left) and ASC (right) expression in the NBP treated, DMSO treated, and sham groups in BV2 culture. Bar graphs showed that NLRP3 (left) and ASC (right). (**D**) Photomicrographes showed that TUNEL positive staining in mixture of culture medium from NBP treated and oil treated groups of BV2. Bar graph showed that the number of TUNEL positive cells in these 2 deferent treatments. Scale Bar = 100 μm. N = 3–5 per group. Data were presented as mean ± SD. * *p* < 0.05, ** *p* < 0.01 compared to DMSO group. # *p* < 0.05, ## *p* < 0.01, ### *p* < 0.001 compared to SHAM group. Full pictures of the Western blots and the densitometry scans are presented in Figure S3.

Neuronal death is enhanced or triggered by nearby neurons or glial cells after brain injury [33]. To explore the relationship between NBP-treated microglia and neurons, we performed in vitro experiments (Figure 5A). First, we assessed the survival rate of BV2 cells after OGD with different NBP concentrations by CCK8. We found that the survival rate of BV2 cells after OGD plateaued at an NBP concentration of 70 µM (*p* < 0.05; Figure 5B). Consequently, the BV2 cells were treated with 70 µM NBP in subsequent experiments. We found significantly lower NLRP3 and ASC levels with NBP treatment of BV2 cells after OGD (*p* < 0.05; Figure 5C). When neurons were treated with the BV2 cell cultured medium, neuronal apoptosis was reduced in the NBP-treated compared to the DMSO-treated cell culture medium (*p* < 0.05; Figure 5D).

## 4. Discussion

This study has shown that NBP reduces infarct volume and improves neurobehavioral outcomes in mice following tMCAO. It also attenuated inflammatory factor expression and inhibited microglial NLRP3 inflammasome and neuronal CASP1 expression. Moreover, NBP indirectly reduced neuronal apoptosis in vitro. Our results indicate that NBP attenuates ischemia-induced inflammatory responses in animals, suggesting the multiple effects of NBP are not limited to acute neuroprotection but also attenuation of inflammatory responses.

NBP is a compound extracted from celery seed approved by the China Food and Drug Administration for treating acute ischemic stroke [18]. NBP has shown excellent anti-inflammatory effects in animal models of many brain diseases, including Alzheimer’s disease (AD), Parkinson’s disease (PD), multiple sclerosis (MS), amyotrophic lateral sclerosis (ALS), traumatic brain injury (TBI), and ischemic stroke [30]. The BBB only permits low molecular weight (<400–500 Da) and small lipophilic molecules to enter the brain [34]. NBP is a lipophilic compound with low molecular weight (190.24 Da; Figure 1B). Furthermore, the BBB is compromised after tMCAO in our experiment, suggesting that NBP can cross the BBB after oral administration.

There was a strong inflammatory response in brain tissues after ischemic stroke. Our results show that NBP treatment can reduce IL-1β, TNF-α, IL-6, and other cytokine and chemokine mRNA levels on day 3 after tMCAO. Since inflammatory signaling is a key factor in many neurodegenerative diseases, targeting inflammatory factor overexpression is considered a potential therapeutic approach for these diseases. The NLRP3 inflammasome is a key upstream inflammatory response signal after ischemic stroke [35]. The NLRP3 inflammasome contains three domains: a nucleotide-binding and oligomerization domain (NACHT), an N-terminal pyrin domain (PYD), and a leucine-rich repeat domain (LRR). Normally, the NACHT binds to the LRR, inactivating NLRP3. However, after ischemia, decreased blood flow and parenchymal cell damage due to hypoxia induces NLRP3 inflammasome assembly with ASC and CASP1 leading to a complex cascade of events, resulting in further cell death and a severe inflammatory response [36]. After ischemic stroke, necrotic tissue due to this process is released into the extracellular space as DAMPs. DAMPs then activated the NLRP3 inflammasome in parenchymal cells, leading to further inflammatory responses and tissue damage.

NBP reduced the pathological AD symptoms by inhibiting NLRP3 inflammasome activation [23]. In addition, NBP inhibited NLRP3 inflammasome activation in pMCAO rats, and molecular docking analyses showed that NBP binds to NLRP3 and CASP1 [24]. NBP therapy also inhibited the NLRP3 inflammasome in PD [37]. We found that NBP reduced protein levels of NLRP3, ASC and CASP1. These results indicate that NBP treatment during the acute stage after ischemic stroke is a potential approach for reducing inflammasome activity and its subsequent cascade of events after tMCAO. The NBP doses provided to mice were calculated based on clinical guidelines. Clinically, 800 mg of NBP is administered orally daily [38]. The equivalent dose ratio of mice to humans is 9.1, so the mice were provided 100 mg/kg of NBP.

NLRP3 inflammasomes exist in various cells. The NLRP3 inflammasome in the CNS is mainly found in microglia [10], which are often regarded as resident mononuclear phagocytes involved in CNS neuroinflammation [10]. However, when microglia phagocytose ASC, it leads to microglia activation and pyroptosis [39,40]. ASC specks are structurally similar to prions and can spread inflammation through blood vessels [41]. Our results show that ASC protein is mainly expressed in microglia 3 days after tMCAO, and NBP significantly reduces the number of ASC^+^ microglia. Pyroptosis is a programmed cell death process dependent on CASP1 [15]. We found that NBP significantly reduces neuron and CASP1^+^ neuronal death on 3 days after stroke, suggesting that NBP treatment partially reduces neuronal death caused by pyroptosis.

Our study highlighted the relationship between NBP and the NLRP3 inflammasome in vivo and in vitro. However, due to the lack of a commercial NLRP3-specific agonist, our study does not provide direct evidence that NBP’s neuroprotective effects are mediated by NLRP3 [42]. However, stroke often co-occurs with diseases related to aging. Elderly mice with diabetes, hypertension, and other diseases are required to study its co-occurrence with other clinical diseases in the future.

In summary, NBP reduces the expression of inflammasome-associated proteins and ASC in microglia, decreasing the inflammatory response after tMCAO, reducing neuronal death, and eventually improving neurological function recovery.

## Figures and Tables

**Table 1:** Primers used in this study.

Primer	Sequence (5′-3′)
TNF-α-F	TAGCCAGGAGGGAGAACAGA
TNF-α-R	CCAGTGAGTGAAAGGGACAGA
IL-1α-F	CGCTTGAGTCGGCAAAGAAAT
IL-1α-R	CTTCCCGTTGCTTGACGTTG
IL-1β-F	TACATCAGCACCTCACAAGC
IL-1β-R	AGAAACAGTCCAGCCCATACT
GAPDH-F	AAATGGTGAAGGTCGGTGTG
GAPDH-R	AGGTCAATGAAGGGGTCGTT
IL-6-F	ACCAAGACCATCCAATTCATC
IL-6-R	CTGACCACAGTGAGGAATGTC
IL-17-F	GTTCGTGCTATTGATTTTCAGC
IL-17-R	GGACCCCTTTACACCTTCTTT
IL-18-F	AGGACACTTTCTTGCTTGCCA
IL-18-R	CACAAACCCTCCCCACCTAAC

## Data Availability

The data of the study are available from the corresponding authors upon reasonable request.

## Supplementary Materials

The following supporting information can be downloaded at: https://www.mdpi.com/article/10.3390/life12081244/s1.
