# Supplementary material for: Dl-3-n-Butylphthalide Reduced Neuroinflammation by Inhibiting Inflammasome in Microglia in Mice after Middle Cerebral Artery Occlusion
Source: Life (Basel). 2022 Aug 16;12(8):1244. doi: 10.3390/life12081244 (PMC9410391; doi:10.3390/life12081244)
Supplement: Supplementary file 1 [file life-12-01244-s001.zip › life-1779606-supplementary.pdf]

The data from top to bottom in the table corresponds to the image from left to right.

**Figure S1.**

The Western blot results of NLRP3.

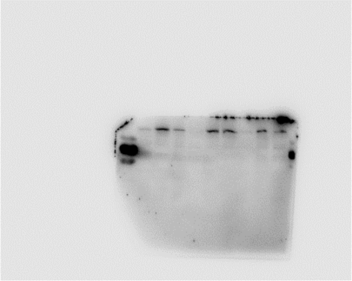
(NLRP3)

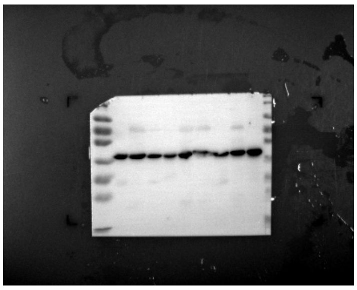
(actin)

| NLRP3 | actin | ratio | Group |
|---|---|---|---|
| 31291 | 82434 | 0.379589 | SHAM |
| 57909 | 96433 | 0.60051 | OIL |
| 39561 | 67965 | 0.582079 | NBP |
| 30454 | 60578 | 0.502724 | SHAM |
| 55106 | 94028 | 0.586059 | OIL |
| 57804 | 96498 | 0.599018 (An unsuccessful detection) | NBP |
| 35449 | 72790 | 0.487004 | SHAM |
| 53276 | 99016 | 0.538054 | OIL |
| 58307 | 126130 | 0.462277 | NBP |

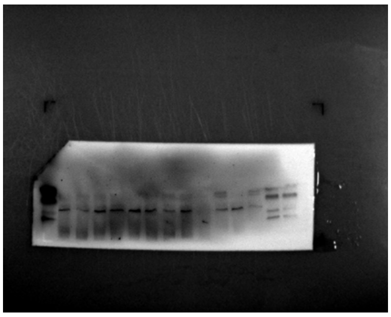
(NLRP3)

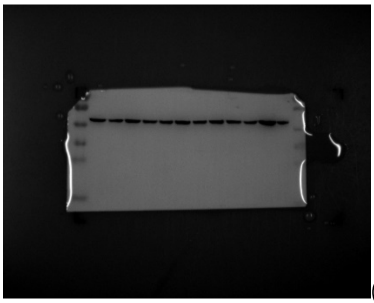(β-actin)

| NLRP3 | actin | ratio | Group |
|---|---|---|---|
| 32432 | 42598 | 0.76135 | OIL |
| 28506 | 39648 | 0.718977 | OIL |
| 37931 | 49481 | 0.766577 | OIL |
| 50738 | 39306 | 1.290846 | OIL |
| 45475 | 41149 | 1.10513 | OIL |
| 39750 | 50628 | 0.785139 | OIL |
| 30616 | 41929 | 0.730187 | NBP |
| 32391 | 49781 | 0.65067 | NBP |
| 22964 | 44536 | 0.515628 | NBP |
| 32066 | 42334 | 0.757453 | NBP |
| 36231 | 78563 | 0.461171 | NBP |
| 26267 | 40747 | 0.644636 | NBP |

## The Western blot results of ASC.

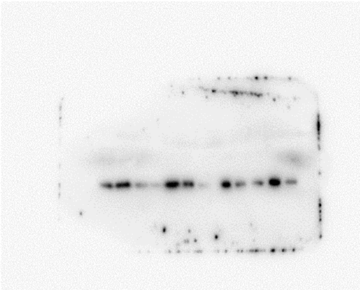

（ASC）

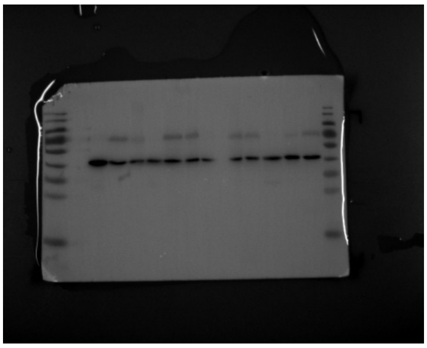

（β-actin）

| ASC | β-actin | ratio | group |
|---|---|---|---|
| 60563 | 99346 | 0.609617 | SHAM |
| 94195 | 64558 | 1.459076 | OIL |

| | | | |
|---|---|---|---|
| 49048 | 54315 | 0.903029 | NBP |
| 31026 | 57275 | 0.541702 | SHAM |
| 104141 | 66760 | 1.559931 | OIL |
| 73824 | 56991 | 1.295362 | NBP |
| 29503 | 37449 | 0.787818 | SHAM |
| 82181 | 42525 | 1.932534 | OIL |
| 55189 | 51223 | 1.077426 | NBP |
| 52401 | 59945 | 0.874151 | SHAM |
| 104075 | 65802 | 1.581639 | OIL |
| 51679 | 65632 | 0.787406 | NBP |

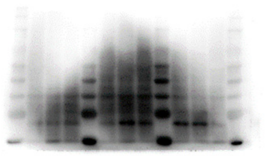

（Photographs of ASC. We used the middle three bands as a schematic diagram, so there was no statistical analysis.）

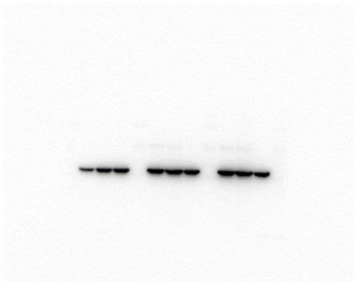

（β-actin）

**Figure S2**

The Western blot results of caspase-1.

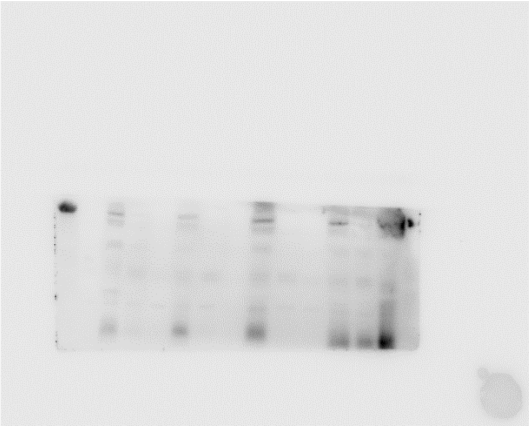

(caspase-1 and cleaved caspase-1)

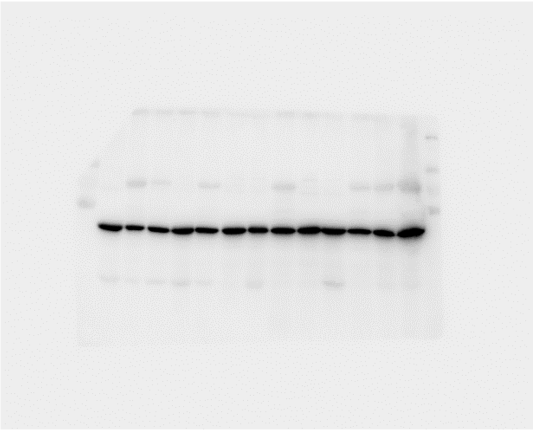

(β-actin)

| CASP1 | β-actin | Ratio1 | Group |
|---|---|---|---|
| 15263 | 97499 | 0.156545 | SHAM |
| 27013 | 70225 | 0.384664 | OIL |
| 18872 | 77812 | 0.242533 | NBP |
| 16835 | 89528 | 0.188042 | SHAM |
| 25010 | 88034 | 0.284095 | OIL |
| 18696 | 108294 | 0.172641 | NBP |
| 17788 | 85985 | 0.206873 | SHAM |
| 39385 | 112325 | 0.350634 | OIL |
| 18653 | 93816 | 0.198825 | NBP |
| 17524 | 115658 | 0.151516 | SHAM |
| 34517 | 103691 | 0.332883 | OIL |
| 26554 | 102721 | 0.258506 | NBP |
| 68801 | 157540 | 0.436721 | OIL |

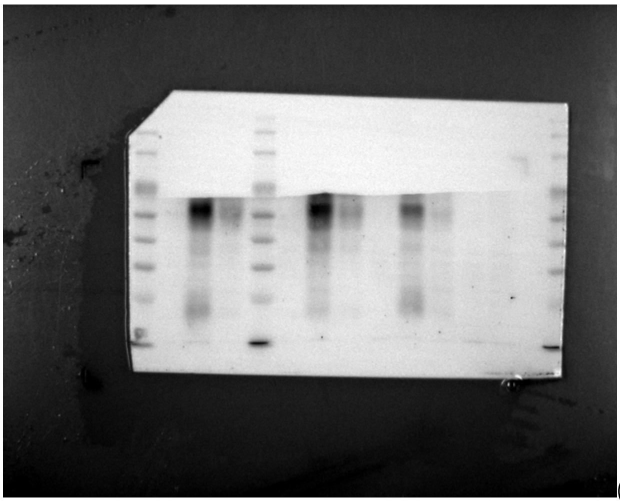(Photographs of caspase-1 and cleaved caspase-1. We used the left three bands as a schematic diagram, so there was no statistical analysis.)

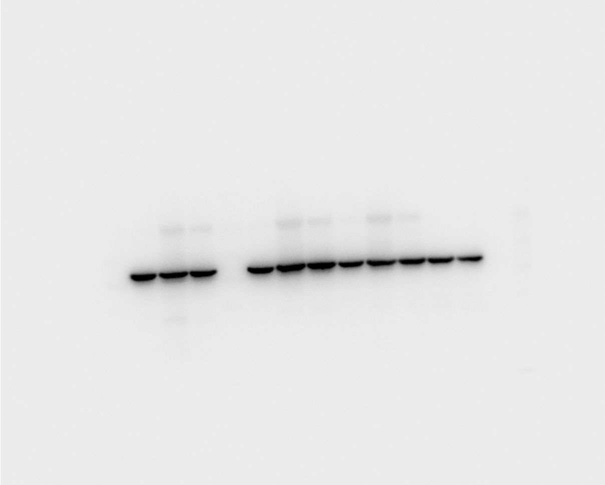

（β-actin）

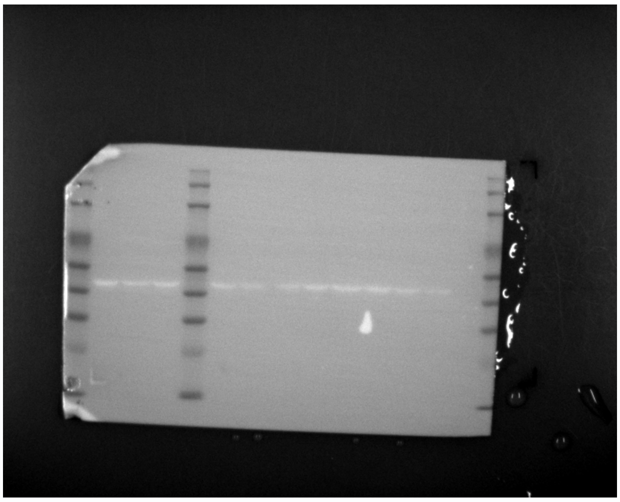

（Bright field images）

## Figure S3

The Western blot results of NLRP3 and ASC from BV2.  The first time.

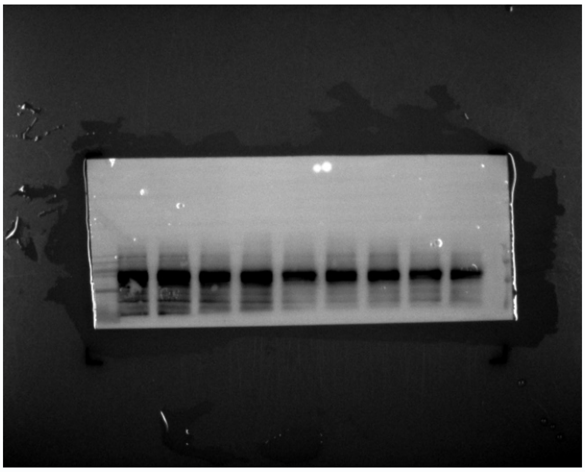

（NLRP3）

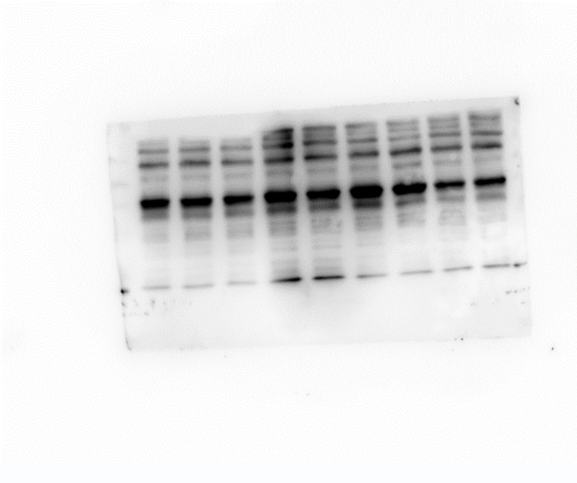

(ASC)

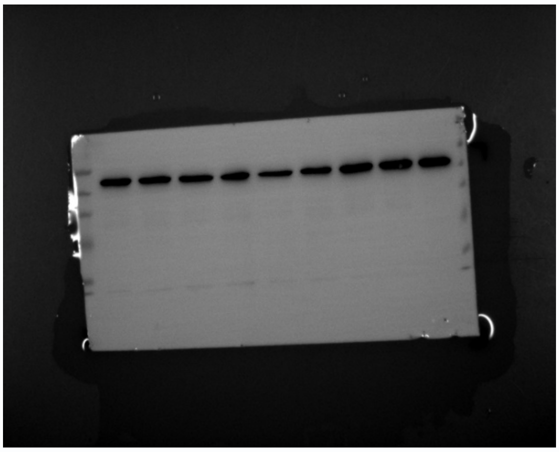

(β-actin)

| NLRP3 | ASC | β-actin | Ratio of NLRP3 | Ratio of ASC | Group |
|---|---|---|---|---|---|
| 211317 | 181009 | 189383 | 1.115818 | 0.955783 | SHAM |
| 241657 | 187023 | 198921 | 1.214839 | 0.940187 | SHAM |
| 217226 | 182478 | 196801 | 1.103785 | 0.927221 | SHAM |

| | | | | | |
|---|---|---|---|---|---|
| 228660 | 226170 | 186139 | 1.228437 | 1.21506 | DMSO |
| 203886 | 215707 | 158058 | 1.289944 | 1.364733 | DMSO |
| 207295 | 229780 | 176764 | 1.172722 | 1.299925 | DMSO |
| 201670 | 201169 | 227495 | 0.886481 | 0.884279 | NBP |
| 189832 | 153177 | 234792 | 0.808511 | 0.652394 | NBP |
| 174735 | 157748 | 223973 | 0.780161 | 0.704317 | NBP |

The Western blot results of NLRP3 and ASC from BV2. The second time.

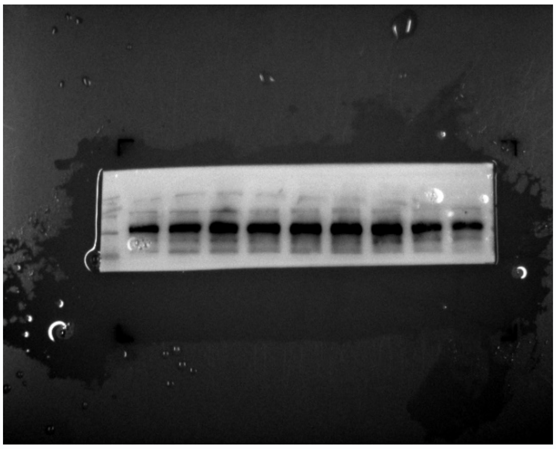
(NLRP3)

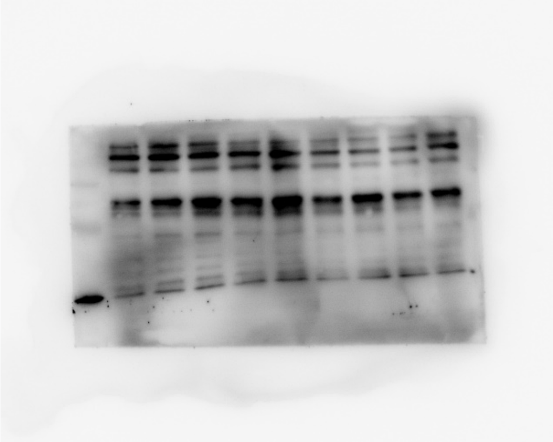
(ASC)

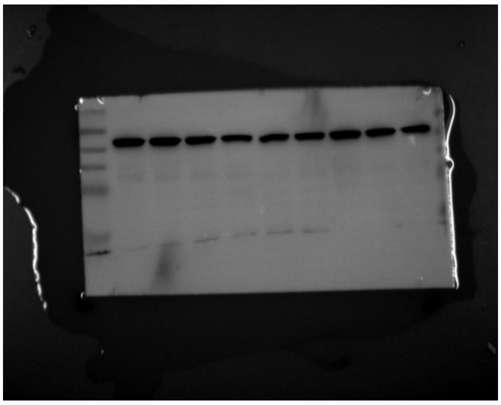

(β-actin)

| NLRP3 | ASC | β-actin | Ratio of NLRP3 | Ratio of ASC | Group |
|---|---|---|---|---|---|
| 181653 | 131781 | 190873 | 0.951696 | 0.690412 | SHAM |
| 215205 | 155746 | 191010 | 1.126669 | 0.815381 | SHAM |
| 232245 | 167881 | 173485 | 1.338704 | 0.967697 | SHAM |
| 235388 | 167962 | 138232 | 1.702847 | 1.215073 | DMSO |
| 239363 | 179275 | 138330 | 1.730377 | 1.295995 | DMSO |
| 239205 | 133934 | 143713 | 1.664463 | 0.931955 | DMSO |
| 247638 | 157818 | 188026 | 1.317041 | 0.839341 | NBP |
| 214068 | 136283 | 171503 | 1.248188 | 0.794639 | NBP |
| 185876 | 147712 | 152813 | 1.216362 | 0.966619 | NBP |

The Western blot results of NLRP3 and ASC from BV2. The third time.

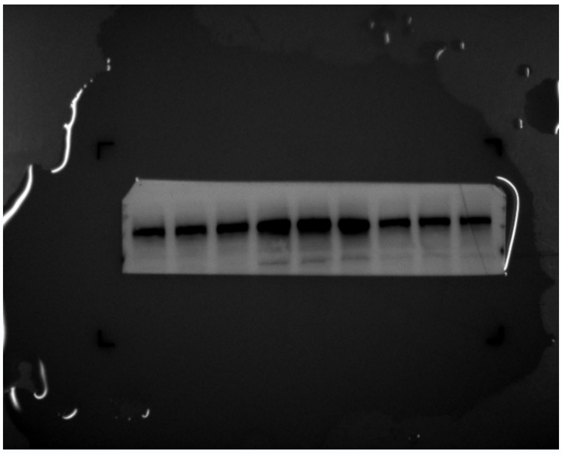

（NLRP3）

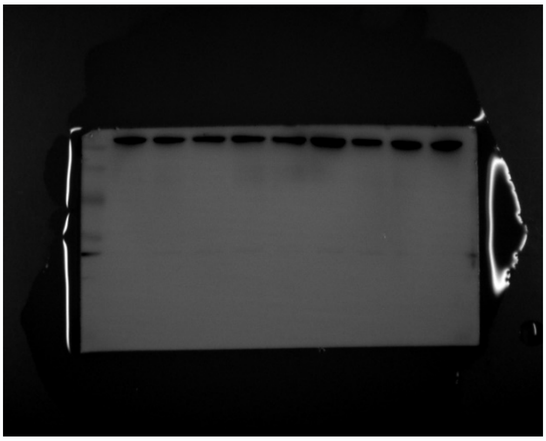
（β-actin）

| NLRP3 | β-actin | Ratio of NLRP3 | Group |
|---|---|---|---|
| 186584 | 178550 | 1.044996 | SHAM |
| 196879 | 163638 | 1.203137 | SHAM |
| 205608 | 163834 | 1.254978 | SHAM |
| 247613 | 177074 | 1.398359 | DMSO |
| 258660 | 187270 | 1.381214 | DMSO |
| 246722 | 223382 | 1.104485 | DMSO |
| 187945 | 178370 | 1.053681 | NBP |
| 190528 | 191423 | 0.995324 | NBP |
| 180700 | 193246 | 0.935078 | NBP |

The Western blot results of NLRP3 and ASC from BV2. The fourth time.

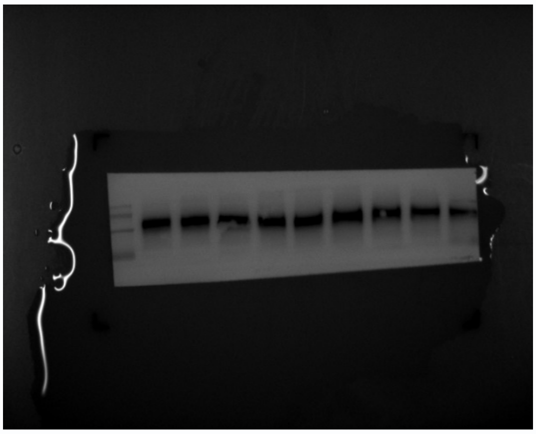
（NLRP3）

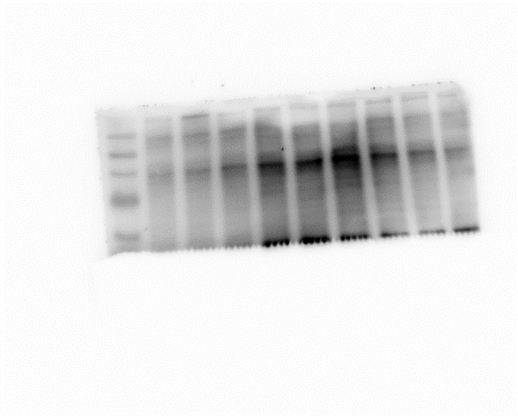

（ASC ）

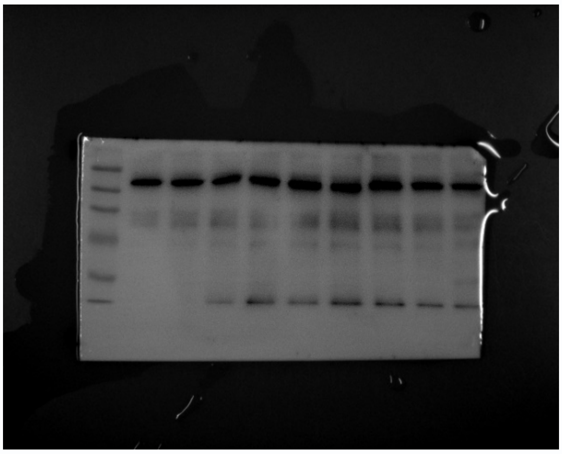

（actin）

| NLRP3 | ASC | β-actin | Ratio of NLRP3 | Ratio of ASC | Group |
|---|---|---|---|---|---|
| 202719 | 69993 | 163749 | 1.237986 | 0.427441 | SHAM |
| 213827 | 88350 | 181037 | 1.181123 | 0.488022 | SHAM |
| 197053 | 111148 | 179017 | 1.10075 | 0.62088 | SHAM |
| 207441 | 148370 | 197098 | 1.052476 | 0.752773 | DMSO |
| 219704 | 158517 | 220379 | 0.996937 | 0.719293 | DMSO |
| 234634 | 180038 | 220406 | 1.064554 | 0.816847 | DMSO |
| 201426 | 146583 | 227133 | 0.88682 | 0.645362 | NBP |
| 192567 | 105058 | 192811 | 0.998735 | 0.544876 | NBP |
| 153373 | 78878 | 173095 | 0.886063 | 0.455692 | NBP |

The WHOLE blot of NLRP3

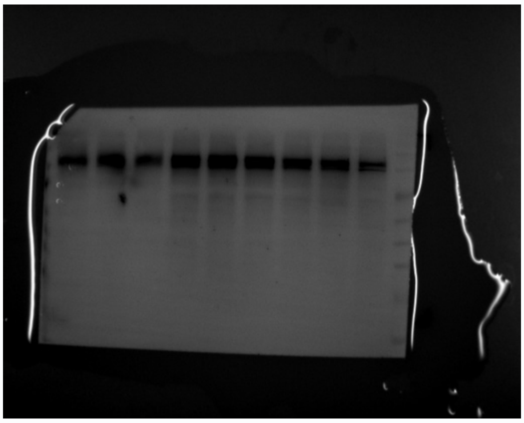

（NLRP3）

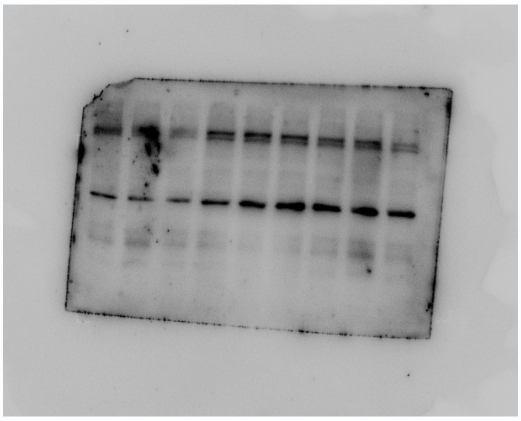

（β-actin）

| NLRP3 | β-actin | Ratio of NLRP3 | Group |
|---|---|---|---|
| 201654 | 195023 | 1.034001 | SHAM |
| 227505 | 193667 | 1.174723 | SHAM |
| 193979 | 174245 | 1.113254 | SHAM |
| 239596 | 200142 | 1.19713 | DMSO |
| 247479 | 211090 | 1.172386 | DMSO |
| 238710 | 235006 | 1.015761 | DMSO |
| 219569 | 225074 | 0.975541 | NBP |
| 218933 | 235514 | 0.929597 | NBP |
| 172273 | 214474 | 0.803235 | NBP |
